# Assessment of national dosimetry quality audits results for teletherapy machines from 1989 to 2015

**DOI:** 10.1120/jacmp.v17i2.5984

**Published:** 2016-03-08

**Authors:** Wazir Muhammad, Asad Ullah, Khalid Mahmood, Matiullah

**Affiliations:** ^1^ Health Physics Division (HPD) Pakistan Institute of Nuclear Science and Technology (PINSTECH) Islamabad 45650 Pakistan; ^2^ Directorate of Systems and Services Pakistan Institute of Nuclear Science & Technology (PINSTECH) Islamabad 45650 Pakistan

**Keywords:** radiotherapy, radiation dosimetry, dosimetry quality audit, on‐site dosimetry visits

## Abstract

The purpose of this study was to ensure accuracy in radiation dose delivery, external dosimetry quality audit has an equal importance with routine dosimetry performed at clinics. To do so, dosimetry quality audit was organized by the Secondary Standard Dosimetry Laboratory (SSDL) of Pakistan Institute of Nuclear Science and Technology (PINSTECH) at the national level to investigate and minimize uncertainties involved in the measurement of absorbed dose, and to improve the accuracy of dose measurement at different radiotherapy hospitals. A total of 181 dosimetry quality audits (i.e., 102 of Co‐60 and 79 of linear accelerators) for teletherapy units installed at 22 different sites were performed from 1989 to 2015. The percent deviation between users’ calculated/stated dose and evaluated dose (in the result of on‐site dosimetry visits) were calculated and the results were analyzed with respect to the limits of ±2.5% (ICRU “optimal model”) ±3.0% (IAEA on‐site dosimetry visits limit) and ±5.0% (ICRU minimal or “lowest acceptable” model). The results showed that out of 181 total on‐site dosimetry visits, 20.44%, 16.02%, and 4.42% were out of acceptable limits of ±2.5%±3.0%, and ±5.0%, respectively. The importance of a proper ongoing quality assurance program, recommendations of the followed protocols, and properly calibrated thermometers, pressure gauges, and humidity meters at radiotherapy hospitals are essential in maintaining consistency and uniformity of absorbed dose measurements for precision in dose delivery.

PACS number(s): 87.50.cm, 87.50.sj, 87.50.up, 87.50.wj, 87.50.yk, 87.55.km, 87.55.Qr

## I. INTRODUCTION

Radiation therapy has an increasingly important role in the medical field, particularly in the treatment of malignant diseases, such as cancer. Worldwide, about 40% of cancer patients require radiation treatment, either curative or palliative.^1^, ^2^ In this regard, radiation therapy is the only application of radiation in which very high radiation doses are deliberately delivered to a human body. However, maximum control on radiation dose to the target area of an individual (with minimum effects to the normal tissues) depends on various factors, particularly the accuracy in delivering the intended or prescribed dose. Severe toxicities or even fatal consequences can emerge from a slight variation from prescribed dose and can lead to an accidental consequences.^3^, ^4^ To prevent such unwanted events, a recommendation of at least ±5% accuracy or prescription at the 95% confidence level in the delivery of absorbed dose to the target volume of the treatment tumor has been suggested.^5^, ^6^, ^7^, ^8^, ^9^ These recommendations in clinical dosimetry are achieved gradually by assessing clinical data. However, radiotherapy is a multistep process and uncertainties in each step accumulate and affect the final dose delivered to the patients. These recommendations of accuracy level are for the final dose delivered to the patients. Besides these recommendations, Brahme^(10)^ and Mijnheer et al.^(11)^ have proposed a tolerance value of accuracy in dose delivery of ±3.5% and ±3%, respectively, at one standard deviation (SD) level and by considering limiting uncertainties for acceptable increase in normal tissue complication risk.^(5)^ ICRU Report 24 also states the recommended uncertainty in the delivered dose to a phantom at 2.5% (optimal model) and 5% (minimal or “lowest acceptable” model).^(7)^

In radiation therapy, the outcome of the treatment greatly depends on accuracy of the dose delivery to the patients, which in turn depends on target area localization, radiation dosimetry, and treatment planning and patient positioning. Among these factors, radiation dosimetry has a vital role in accurate dose delivery to the target area of the patients. However, published data^4^, ^5^, ^8^ have showed that many radiotherapy units have not been used properly because of inadequate dosimetry audits and lack of proper error analysis. Literature has showed that a total of 2,500,000 patients are yearly treated by around 6,000 to 7,000 teletherapy (i.e., Co‐60 and megavolts X‐ray) units. Of these patients, more than 10% are either overexposed or underexposed due to lack of proper equipment, personnel skill or training.^(12)^ In order to circumvent these problems and achieve the required level of accuracy (as stated earlier) and maintain consistency, a continued and comprehensive quality assurance (QA) of each step of radiotherapy is essential according the international guidelines.^13^, ^14^, ^15^ However, an independent external quality audit along with the local QA program is also recognized as a part of an effective method of checking the quality and accuracy in radiation dose delivery to the patients.^(8)^ In addition, this also establishes greater confidence in dose delivery among the local radiotherapy community. Due to the importance of the subject, the local and international regulatory organizations also emphasized the independent external quality audits. These organizations compelled the individual radiotherapy hospitals through their regulations to perform the audit on regular basis. The dosimetry audits and errors traceability of relevant clinics may be achieved either by postal measurements (i.e., by mailed‐in dosimeters), on‐site dosimetry visits using ionization chambers,^(5)^ or a mixture of both. Even though the on‐site dosimetry visits are troublesome because of traveling of personnel, equipment, and financial constraints, yet it is the most efficient and important method, as the dosimetry data and techniques are reviewed objectively. Furthermore, all the interfering parameters can be easily traced out on the spot and, as a result, the remedial actions can be immediately discussed with the local medical physicist at the radiotherapy hospital.^(16)^ For this task, the best suited organization may be either Primary Standards Dosimetry Laboratories (PSDLs) or Secondary Standards Dosimetry Laboratories (SSDLs) of the country, in addition to provided calibrated dosimetry systems at the relevant radiotherapy hospitals.^(17)^ As these laboratories have the standard/calibrated radiation dosimetry systems/sources and generally have trained/skilled manpower. For postal measurements and on‐site dosimetry visits to radiotherapy hospitals, the uncertainties should not be out of the range ±5% and ±3%, respectively.^9^, ^18^, ^19^, ^20^

In view of the above‐mentioned factors, an external dosimetry quality audit program has been designed at national level by the national SSDL through on‐site dosimetry visits to radiotherapy hospitals, starting in 1989. Since then, yearly, radiation beam output of teletherapy machines installed at different radiotherapy hospitals of Pakistan has been carried out. This study summarized and analyzed the results of these countrywide on‐site dosimetry visits to the radiotherapy centers. The study is also designed to discuss the possible sources of errors and provide suggestions to reduce these errors.

## II. MATERIALS AND METHODS

A series of dosimetry quality audits based on‐site visit with a reference dosimetry system, were performed for teletherapy units installed in different radiotherapy centers nation‐wide. According to the recommendations for on‐site dosimetry quality audit procedures, an external dosimetry system should be used for the audit. The dosimetry system (used for the on‐site dosimetry quality audits) consisted of a Farmer‐type cylindrical ionization chamber model NE2571 (Nuclear Enterprises America, Fairfield, NJ) with active volume of 0.69 cm^3^)^(21)^ connected to an electrometer (Farmer‐type dosimeter NE2570).^(22)^ The dosimetry system was attached to an IAEA stationary water phantom of size 30 cm×30 cm×30 cm. The dosimetry system was calibrated in a Co‐60 radiation beam at Secondary Standard Dosimetry Laboratory (SSDL) at

PINSTECH, Pakistan, following IAEA TRS‐398 protocol. The calibration (both in terms of air kerma and absorbed dose to water) was performed against a dosimetry system (calibrated both in terms of air kerma and the absorbed dose to water against the primary standard of the IAEA dosimetry laboratory, Vienna, Austria) consisting of an ionization chamber (type NE2561/NPL, serial no. 200) connected to an electrometer (type 2560, serial no. 173).^(11)^ Apart from the above dosimetry system and phantom, three barometers (Sr. No. B823, 86947, and 95882) and two thermometers (2100 Tele, Sr. No. 662, and glass thermometer, Sr. 57/15) were also used in order to make pressure and temperature corrections to the ionization chamber readings. Both the barometer and thermometer were calibrated from National Physical Standard Laboratory (NPSL), Islamabad, which is a subdivision of Pakistan Council of Scientific and Industrial Research (PCSIR).

All measurements were performed following the reference conditions of IAEA TRS‐277 and IAEA TRS‐398 dosimetry protocol (i.e., TRS‐277 was followed before the implementation of TRS‐398).^23^, ^24^ By following TRS‐277, the chamber was aligned in air at the reference depth from the source with buildup cap for a field size of 10 cm×10 cm. To follow TRS‐398, the chamber was kept in PMMA sleeve (3.45 mm) and was then aligned in water phantom at the depth of 5 cm from the isocenter with buildup cap for a field size of 10 cm×10 cm.^25^, ^26^ After taking the measurements, the physicists of the visited center were asked to carry out their dosimetry measurements by using their local dosimetry systems and setups (used for their routine dosimetry) at 5 cm depth from the isocenter for a field size of 10 cm×10 cm. After getting their results, % deviation (% Δ) between the “true” dose value (measured with the reference visiting dosimetry system), $D_{T}$, and the requested local dose determination (measured contextually with the local dosimetry system), $D_{L}$, was determined according to the following formula:^(19)^

(1) $$\%\Delta =\frac{D_{L}-D_{T}}{D_{T}}\times 100$$

The analyses of the results were performed with respect to the uncertainty limits of ±2.5% (ICRU “optimal model”),^(7)^ ±3.0% (IAEA on‐site dosimetry visits limit),^9^, ^18^, ^27^ and ±5.0% (ICRU minimal or “lowest acceptable” model).^(7)^

## III. RESULTS AND DISCUSSION

**Figure 1:**
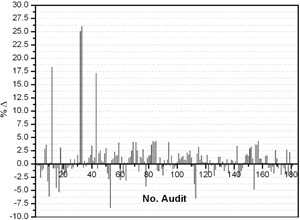
Percentage deviation between the absorbed doses determined by the local physicists and the audit team of SSDL as function of number of audit at reference conditions from 1989 to 2015.

**Figure 2:**
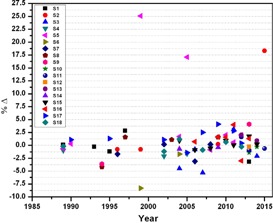
Percentage deviation between the absorbed doses determined by the local physicists and the audit team of SSDL at reference conditions for Co‐60 teletherapy units from 1989 to 2015.

**Figure 3:**
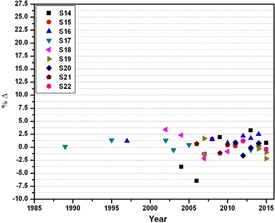
Percentage deviation between the absorbed doses determined by the local physicists and the audit team of SSDL at reference conditions for 6 MV photon beam emitted from linear accelerators from 1989 to 2015.

**Figure 4:**
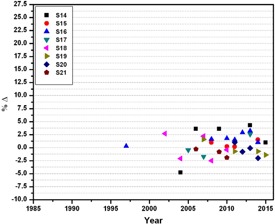
Percentage deviation between the absorbed doses determined by the local physicists and the audit team of SSDL at reference conditions for 10/15 and 18 MV photon beam emitted from linear accelerators from 1989 to 2015.

A total of 181 dosimetry quality audits of teletherapy units (i.e., 102 of Co‐60 and 79 of linear accelerators), installed at 22 different clinics (details are given in Table 1), were performed from 1989 to 2015, as shown in Fig. 1. During the audit, the %Δ between the users stated/measured dose output and the measured dose output (i.e., in the result of on‐site dosimetry visits) was determined. The maximum, minimum, and mean of %Δ for all 181 dosimetry quality audits are ±26.03%, ±0.03%, and ±2.08% respectively with standard deviation of ±3.3. Out of 22 radiotherapy hospitals, six (27.3%) and one (4.5%) have a mean %Δ greater than the acceptable limit of ±2.5% or ±3% and ±5%, respectively, for both Co‐60 units and linear accelerators (see Tables 2 and 3). For Co‐60 teletherapy units, a total of 102 audits were conducted for a total of 18 clinics. The %Δ as function of year is plotted in Fig. 2, and also summarized in Table 2. Out of these 102 audits for Co‐60 units, 24.5%, 19.6%, and 6.9% are out of acceptable limits of ±2.5%, ±3.0%, and ±5.0%, respectively, as shown in Fig. 2. For linear accelerators, a total 79 audits were conducted for total of nine radiotherapy hospitals (Table 1) and %Δ as function of year is plotted in Figs. 3 and 4 and also summarized in Table 3. Out of 79 on‐site dosimetry audits, 15.2%, 11.4%, and 1.3% were out of acceptable limits of ±2.5%, ±3.0%, and ±5.0%, respectively, as shown in Figs. 3 and 4. Out of all 181 audits, a larger %Δ (>15%) has also been observed for four audits (i.e., 26.03%, 25.08%, 18.3%, and 17.1%). These cases were thoroughly assessed and reasons were explored and conveyed to the users to minimize the errors. However, some common reasons are discussed here for the guidance of the readers. In most of these cases, correction of air density (pressure and temperature correction factor) might be the great cause because most of the radiotherapy hospitals used uncalibrated barometers and thermometers. According to TRS‐398 dosimetry protocol, thermometers and pressure gauges should be properly calibrated from a relevant calibration laboratory. Secondly, the problems were related to mechanical instability of the machines. These include isocentric deviation, difference between mechanical and optical SSD indicator and digital and mechanical display of the gantry angle. The hospital physicists always preferred to use optical SSD indicator and digital display of the gantry angles and, due to the considerable deviation between the optical and mechanical SSD indicator and digital and mechanical display of the gantry angles, the uncertainty raised. In short, the problems were related to the alignment of dosimetry systems and correct positioning of the ionization chambers. In some cases, mistakes in the implementation of the dosimetry protocols were also found (i.e., in using the correction factors, $K_{\text{qqo}},K_{\text{pol}},K_{\text{sat}}$). However, it is worth mentioning here that the dosimetry systems of the audited radiotherapy hospitals are calibrated from the Secondary Standard Dosimetry Laboratory (SSDL), Pakistan, which is a part of IAEA/WHO SSDL network.

**Table 1:** Summary of the nation‐wide on‐site dosimetry visits of radiotherapy hospitals from 1989 to 2015

		*Radiotherapy Unit Audited*			*No. of Audits*		
*S. No*.	*Code*	*Co‐60 Unit*	*Linear Accelerator*	*No. of Audits*	≥±2.5	≥±3.0	≥±5.0
1.	S1	√	x	10	05	03	01
2.	S2	√	x	04	01	01	01
3.	S3	√	x	05	03	03	01
4	S4	√	x	03	‐	‐	‐
5.	S5	√	x	19	04	04	03
6.	S6	√	x	03	02	01	01
7.	S7	√	x	04	01	01	‐
8.	S8	√	x	04	01	01	‐
9.	S9	√	x	03	03	03	‐
10.	S10	√	x	02	‐	‐	‐
11.	S11	√	x	03	‐	‐	‐
12.	S12	√	x	02	‐	‐	‐
13.	S13	√	x	02	‐	‐	‐
14.	S14	√	√	11	07	07	01
15.	S15	√	√	15	‐	‐	‐
16.	S16	√	√	22	04	02	‐
17.	S17	√	√	22	04	02	‐
18.	S18	√	√	19	02	01	‐
19.	S19	x	√	11	‐	‐	‐
20.	S20	x	√	08	‐	‐	‐
21.	S21	x	√	06	‐	‐	‐
22.	S22	x	√	02	‐	‐	‐
Total		18	09	181	37 (20.44%)	29 (16.02%)	08 (4.42%)

**Table 2:** Summary of the dosimetry quality audits of Co‐60 teletherapy units installed at 18 different sites (Sn) from 1989 to 2015

			*% Deviation*			
*Site*
$\left(S_{n}\right)$	*Audits Period*	*No. of Audits*	Mean (±)	*Standard Deviation*	Minimum (±)	Maximum (±)
S1	1989‐2013	10	2.11	1.91	0.1	6.1
S2	1996‐2015	4	5.03	8.85	0.2	18.3
S3	2004‐2014	5	3.16	1.81	0.8	5.3
S4	1989‐2005	3	1.57	0.55	1.0	2.1
S5	1989‐2012	19	4.7	8.24	0.3	26.03
S6	1999‐2004	3	4.27	3.54	1.7	8.3
S7	1996‐2008	4	1.3	1.39	0.2	3.1
S8	1994‐2009	4	2.13	1.4	1.1	4.2
S9	1994‐2013	3	4.0	0.36	3.6	4.3
S10	1999‐2012	9	0.96	0.58	0.2	2.1
S11	2013‐2014	2	0.85	0.78	0.3	1.4
S12	2012‐2015	3	1.07	0.49	0.64	1.6
S13	2013‐2014	2	0.35	0.07	0.3	0.4
S14	2012‐2014	2	1.45	0.78	0.9	2.0
S15	2004‐2009	2	0.55	0.21	0.4	0.7
S16	2004‐2014	7	0.75	0.56	0.03	1.73
S17	2006‐2013	8	1.94	1.11	0.7	4.0
S18	1990‐2012	12	1.9	1.3	0.2	4.1

**Table 3:** Summary of the dosimetry quality audits of linear accelerators installed at nine different sites ($S_{n}$) from 1989 to 2015

*Site* $\left(S_{n}\right)$	*Audits Period*	*Beam Energy (MV)*	*No. of Audits*	Mean (±)	*SD*	Minimum (±)	Maximum (±)
S14	2004‐2015	6	5	3.24	2.16	0.8	6.5
		15	5	3.45	1.49	0.95	4.8
S15	2008‐2014	6	4	0.62	0.64	0.2	1.57
		15	4	0.71	0.65	0.16	1.5
S16	1997‐2015	6	7	1.50	0.67	0.7	2.5
		15	7	1.76	1.02	0.28	3.2
S17	1989‐2013	6	7	0.79	0.5	0.11	1.32
		10/15	3	1.6	1.05	0.48	2.56
S18	2002‐2010	6	5	2.01	0.99	0.82	3.4
		10/15	5	1.98	0.91	0.41	2.7
S19	2007‐2015	6	7	1.08	0.75	0.2	2.2
		18	4	1.12	0.45	0.73	1.6
S20	1995‐2014	6	4	0.84	0.62	0.1	1.6
		15	4	0.98	0.79	0.1	2.01
S21	2006‐2010	6	3	0.74	0.37	0.44	1.15
		15	3	1.02	0.83	0.3	1.93
S22	2012‐2015	6	2	0.8	0.57	0.4	1.2

The dosimetry quality audit is very important not only to check the accuracy of the radiation dose delivery to the patients, but also to reveal problems/issues related to the dosimetry measurements. These on‐site visits/audits have fruitful results in providing guidelines regarding implementation of the dosimetry protocols accurately. The highlighted issues/problems as a result of these audits included the unavailability of distilled water, properly calibrated thermometers, and pressure gauges and humidity meters at the radiotherapy hospitals. Moreover, the water was not kept at least 24 hours before the performing the dosimetry measurements at the measurement place for temperature stability. Most of the hospitals physicists were observed not using dosimetry systems and protocols properly. Moreover, it was also observed that physicists at the hospitals were normally not performing the reference check source measurements for their dosimetry systems (internal quality audit of dosimetry system). It was also noted that some of the hospitals followed different dosimetry protocols although their dosimetry systems were calibrated from SSDL, HPD, Pakistan, based on IAEA TRS‐398 dosimetry protocol. The considerable deviation in laser alignment systems with the isocenter of the source, locally fabricated/repaired treatment couch, and mechanical instability of the machines were also among the reasons for the uncertainties. Furthermore, some hospitals were also using Co‐60 units for radiotherapy treatments that had much lower dose rates than the IAEA recommended dose rate (i.e., 40 cGy/min for field size of 10 cm×10 cm at the depth of $D_{\text{max}}$) level.^(27)^

## V. CONCLUSIONS

The deviation from audited dose measurements arose due to the unavailability of calibrated thermometers, pressure gauges, and humidity meters at clinics. Attention was not given to properly following the recommendations/procedures of the relevant protocols about usage of water for measurements. The last main reason was related to issues regarding the quality assurance program of the teletherapy units for radiotherapy. Therefore, the importance of a proper ongoing quality assurance program and recommendations of the followed protocols is essential in maintaining the consistency and the uniformity of absorbed dose measurement for the precision of dose delivery to the patient.

## ACKNOWLEDGMENT

The authors would like to acknowledge helpful technical support and discussions with Ikram Ullah Qazi, Deputy Chief Scientist (Retired), Health Physics Division, Pakistan Institute of Nuclear Science and Technology (PINSTECH), Islamabad 45650, Pakistan.

## COPYRIGHT

This work is licensed under a Creative Commons Attribution 4.0 International License.

## References

[1] Donaldson S . Towards safer radiotherapy. London: British Institute of Radiology, Institute of Physics and Engineering in Medicine, National Patient Safety Agency, Society and College of Radiographers, and The Royal College of Radiologists; 2007.

[2] Frödin JE , Jonsson E , Möller T , Werkö L . Radiotherapy in Sweden — a study of present use in relation to the literature and an estimate of future trends. Acta Radiol Oncol. 1996;35(8): 967–79. 10.3109/02841869609100714 9023381

[3] International Commission on Radiological Protection . Prevention of accidental exposures to patients undergoing radiation therapy. ICRP Publication 86. Ann ICRP. 2000;30(3). 10.1016/s0146-6453(01)00035-5 11711147

[4] Ortiz P . Lessons learned from accidental exposures in radiotherapy. IAEA Safety Reports Series 17. Vienna: IAEA; 2000.

[5] Rahman M , Kim G , Rahman AFM , et al., TLD postal dose quality audit intercomparison for megavolts photon beam of radiotherapy centers in Bangladesh. J Nucl Sci Tech. 2008;45(Supp 5):260–63.

[6] Brahme A, editor. Accuracy requirements and quality assurance of external beam therapy with photons and electrons. Stockholm: Foundation Acta Radiologica; 1988.

[7] International Commission on Radiation Units and Measurements . Determination of absorbed dose in patient irradiated by means of X or gamma rays in radiotherapy procedures. ICRU Report 24. Bethesda, MD: ICRU; 1977.

[8] Thwaites DI . The significance and impact of dosimetry audits in radiotherapy. SSDL Newsletter 58. Vienna: IAEA; 2010.

[9] Izewska J , Dutreix A , Followill DS , et al. Standardized quality audit procedures for on‐site dosimetry visits to radiotherapy hospitals. Report of the IAEA consultants' meeting, IAEA, Vienna, 27 September ‐ 1 October 1999; revised in 2001. SSDL Newsletter 46. Vienna: IAEA; 2002.

[10] Brahme A . Dosimetric precision requirements in radiation therapy. Acta Radiol Oncol. 1984;23(5): 379–91. 6095609 10.3109/02841868409136037

[11] Mijnheer BJ , Battermann JJ , Wambersie A . What degree of accuracy is required and can be achieved in photon and neutron therapy? Radiother Oncol. 1987;8(3):237–52. 3107087 10.1016/s0167-8140(87)80247-5

[12] International Atomic Energy Agency . Report on the fifth meeting of the SSDL Scientific Committee (SSC). SSDL Newsletter 31. Vienna: IAEA; 1992.

[13] Kutcher GJ , Cola L , Gillin M , et al. Comprehensive QA for radiation oncology: report of AAPM Radiation Therapy Committee Task Group 40. Med Phys. 1994;21(4):581–618. 8058027 10.1118/1.597316

[14] Thwaites D , Scalliet P , Leer JW , Overgaard J . Quality assurance in radiotherapy: European Society for Therapeutic Radiology and Oncology advisory report to the commission of the European Union for the ‘Europe Against Cancer Programme.’ Radiother Oncol. 1995;35(1):61–73. 7569014 10.1016/0167-8140(95)01549-v

[15] Klein EE , Hanley J , Bayouth J , et al. Task Group 142 report: quality assurance of medical accelerators. Med Phys. 2009;36(9):4197–212. 19810494 10.1118/1.3190392

[16] Knöös T and Medin J . A dosimetric intercomparison between the radiation therapy clinics in Sweden. Stockholm: Swedish Radiation Safety Authority; 2012.

[17] International Atomic Energy Agency and World Health Organization . Calibration of reference dosimeters for external beam radiotherapy. IAEA Technical Reports Series 469. Vienna: IAEA; 2009.

[18] International Atomic Energy Agency . On‐site visits to radiotherapy centres: medical physics procedures. Vienna: IAEA; 2007.

[19] de Paiva E , da Rosa LA , Brito RR , et al. An analysis of the regulatory program of quality audits in radiotherapy in Brazil from 1995 to 2007. J Appl Clin Med Phys. 2011;12(2):3330. 21587175 10.1120/jacmp.v12i2.3330 PMC5718684

[20] Hourdakis CJ and Boziari A . Dosimetry quality audit of high energy photon beams in greek radiotherapy centers. Radiother Oncol. 2008;87(1):132–41. 18343514 10.1016/j.radonc.2008.01.023

[21] Nuclear Enterprises America . Instruction manual for 0.6 cc ionization chamber type 2571. Fairfield, NJ: Nuclear Enterprises America; 1984.

[22] Nuclear Enterprises America . Instruction manual for 2570A and 2570B Farmer dosimeter. Fairfield, NJ: Nuclear Enterprises America; 1984.

[23] International Atomic Energy Agency . Absorbed dose determination in external beam radiotherapy: an international code of practice for dosimetry based on standards of absorbed dose to water. Technical Reports Series 398. Vienna: IAEA; 2001.

[24] International Atomic Energy Agency . Absorbed dose determination in photon and electron beams: an international code of practice. IAEA Technical Report Series 277, 2 Sub edition. Vienna: IAEA; 1997.

[25] International Atomic Energy Agency . Implementation of the international code of practice on dosimetry in radiotherapy (TRS 398): review of testing results. Vienna: IAEA; 2005.

[26] International Atomic Energy Agency . Review of data and methods recommended in the international code of practice. IAEA Technical Reports Series no. 277, absorbed dose determination in photon and electron beams. Vienna: IAEA; 1996.

[27] International Atomic Energy Agency . Setting up a radiotherapy programme: clinical, medical physics, radiation protection and safety aspects. Vienna: IAEA; 2008.

